# A narrative review of alcohol consumption as a risk factor for global burden of disease

**DOI:** 10.1186/s13011-016-0081-2

**Published:** 2016-10-28

**Authors:** Jürgen Rehm, Sameer Imtiaz

**Affiliations:** 1 Institute for Mental Health Policy Research, CAMH, 33 Russell Street, T505, Toronto, ON M5S 2S1 Canada; 2 Campbell Family Mental Health Research Institute, CAMH, 250 College Street, Toronto, ON M5T 1R8 Canada; 3 Institute of Medical Science (IMS), University of Toronto, Medical Sciences Building, 1 King’s College Circle, Room 2374, Toronto, ON M5S 1A8 Canada; 4 Department of Psychiatry, University of Toronto, 250 College Street, 8th Floor, Toronto, ON M5T 1R8 Canada; 5 Dalla Lana School of Public Health, University of Toronto, 155 College Street, 6th Floor, Toronto, ON M5T 3M7 Canada; 6 Institute for Clinical Psychology and Psychotherapy, TU Dresden, Chemnitzer Str. 46, 01187 Dresden, Germany

**Keywords:** Alcohol, Average level of consumption, Patterns of drinking, Comparative risk assessment, Relative risk, Burden of disease, Cause of death, Global

## Abstract

Since the original Comparative Risk Assessment (CRA) for alcohol consumption as part of the Global Burden of Disease Study for 1990, there had been regular updates of CRAs for alcohol from the World Health Organization and/or the Institute for Health Metrics and Evaluation. These studies have become more and more refined with respect to establishing causality between dimensions of alcohol consumption and different disease and mortality (cause of death) outcomes, refining risk relations, and improving the methodology for estimating exposure and alcohol-attributable burden. The present review will give an overview on the main results of the CRAs with respect to alcohol consumption as a risk factor, sketch out new trends and developments, and draw implications for future research and policy.

## Background

The very first Global Burden of Disease (GBD) Study [1, 2] only gave indications on burden of disease as measured in number of deaths or disability adjusted life years ((DALYs; [3, 4]) by different disease categories. DALYs are a summary gap measure of health combining fatal and non-fatal indicators, specifically summing up the years of life lost due to premature mortality and years of life lost due to disability [5]. The first GBD study was purely descriptive, but a major improvement from the situation in the past, when the sum of the number of deaths claimed by different causes by far exceeded the global number of deaths, even if only numbers within the same organization, such as the World Health Organization (WHO), were added up [1, 6]. The second improvement of this study was that it was not restricted to fatal health outcomes, and with DALYs it included a summary measure combining fatal and non-fatal events [5].

However, already at that stage it became apparent that more information was needed if one wanted to decrease the burden of disease. The concept of risk factor is key here [7]; defined as any attribute, characteristic or exposure of an individual that increases the likelihood of developing a disease or injury [8]. As a consequence, all future GBD studies included Comparative Risk Assessments (CRAs), which estimated the number of deaths and DALYs that could be avoided if certain risk factors were to be eliminated or shifted to a less detrimental distribution [9–12]. Such information was seen as important for health policy, especially in terms of primary prevention ([1, 13]; specifically for alcohol see [14, 15]). Alcohol consumption has always been part of the top 10 risk factors assessed in these CRAs in terms of the attributable global burden of disease.

This review will give an overview on the main results of the CRAs with respect to alcohol consumption, sketch out new trends and developments, and draw implications for future research and policy.

## Alcohol-attributable burden of mortality and disease in the various Comparative Risk Assessments

Table 1 gives an overview of the main CRAs conducted since 1996 on two main outcomes: all-cause mortality as measured in the number of deaths, and burden of disease as measured in DALYs (for a definition of DALYs see [3, 4]).

**Table 1:** Proportion of global mortality and DALYs attributable to alcohol (net burden)

Year	Proportion of global mortality attributable to alcohol	Proportion of global DALYs attributable to alcohol	Reference
1990	1.5 %	3.5 %	[12, 155]
2000	3.2 % (W: 0.6 %; M: 5.6 %)	4.0 % (W: 1.3 %; M: 6.5 %)	[16, 156]
2004	3.8 % (W: 1.1 %; M: 6.3)	4.6 % (W: 1.4 %; M: 7.6 %)	[157]
2010	5.2 % (W: 3.1 %; M: 6.9 %)	3.9 % (W: 2.0 %; M: 5.4 %)	[10, 18]^a^
2012	5.9 % (W: 4.0 %; M: 7.6 %)	5.1 % (W: 2.3 %; M: 7.4 %)	[9]
2013	5.1 % (W: 3.1 %; M: 6.8 %)	4.1 % (W: 1.9 %; M: 5.9 %)	[11, 18]^a^
2015	GBD 2015 and WHO Global Status Report on Alcohol and Health to be published in 2016		

^a^The given estimates for mortality are not available in the Lancet publications of the Global Burden of Disease Study 2010 and 2013 [10, 11]. They were obtained from GDB Compare [18]. Please note that these estimates may change when the methodology changes, so the date of assessment is important

While Table 1 seems to indicate a substantial rise in both alcohol attributable mortality and disease burden, this is not the case. Any CRA estimates depend very much on methodology, and the majority of the variation between the first estimate for 1990 [12] and the other estimates can be explained by the following factors (see also [16, 17]:

- Availability of and methodology used for cause of death and disability statistics on a global level.
- Diseases and causes of death which are seen as causally impacted by alcohol.
- Relative risk estimates used to estimate attributable disease burden.
- Methodology used to derive attributable fractions.

These questions will be discussed separately under different headings below.

Having clarified this, there have been efforts to estimate real changes using the same methodology for comparisons in global or regional alcohol-attributable burden of disease between 1990 and 2010 [10]; between 1990 and 2013 [11, 18] or between for all years from 1990 to 2014 [19, 20].

## Availability of and methodology used for cause of death and disability statistics on a global level

Alcohol has been causally linked to more than 230 ICD 10 three digit disease categories [17], including about 40 that would not exist without alcohol (such as alcohol dependence or alcoholic liver cirrhosis; for a complete list see [21]). However, this does not mean, the various global CRA efforts are including all of these disease categories. First, global health statistics are not that detailed. For most of the population worldwide, there are no vital registries with cause of death information. The World Health Organization (WHO) estimates that vital registries fail to cover about two thirds, or 38 million out of 56 million annual deaths globally [22]. For the rest, i.e., the majority of cause of deaths globally, the basis are verbal autopsies and standardized algorithms to analyze these verbal autopsies and to scale up these results to wider regions [23]. Obviously, the resulting cause of death categories are fewer and broader than the categories of the ICD, as it is impossible to determine detailed cause of deaths via verbal autopsy [24]. As indicated above, burden of disease is composed of mortality and disability. For disability, the data situation is worse than for mortality [25], and estimates are developed based on estimated prevalence of disease categories [26] and disability weights; a disability weight is a factor that reflects the severity of the disease on a scale from 0 (perfect health) to 1 (equivalent to death) [27].

Currently, there are two different organizations which produce estimates for global data on health outcomes: the Institute for Health Metrics and Evaluation (IHME) [28] and the WHO [29]. Methodologies for estimating deaths are overlapping but different, resulting in different estimates on cause of death by categories (e.g., [30]). Secondly, different disability weights are applied for non-fatal outcomes, resulting in different estimates of burden of disease in DALYs ) [31–33]. However, the number of disease categories are the same between both organizations. For CRAs this means, that the number of deaths and DALYs, where attributable fractions are applied to, are different based on which underlying statistics are used. One of the main differences specifically for alcohol consumption as a risk factor are alcohol use disorders, which are defined differently by IHME and WHO, and where different disability weights have been applied (IHME restricted to dependence, WHO including harmful use of alcohol with a non-zero weight). A reconceptualization on heavy drinking over time may help overcome these differences [34].

The availability of only broader categories on a global level has consequences for CRA. Diseases and causes of death like alcoholic cardiomyopathy or alcohol-induced chronic pancreatitis are relevant in many countries (for alcoholic cardiomyopathy: [35]; for pancreatitis: [36]), but there are no global statistics. As a consequence, the impact of alcohol can only indirectly be assessed by using larger categories such as cardiomyopathy or pancreatitis, for which global estimates exist. Unfortunately, often risk relations are missing for such larger categories: this is the case for cardiomyopathy, as there is neither a systematic review nor a meta-analyses for the impact of alcohol, and thus, this category will show no alcohol-attributable cases, even though we know, there are cases of alcoholic cardiomyopathy. For alcohol-induced chronic pancreatitis, the CRA has to rely on the larger category of pancreatitis and meta-analyses on the impact of alcohol on this category [37, 38] to be included into alcohol-attributable deaths or burden of disease. As a consequence, the alcohol-attributable disease and cause of death categories boil down to a much lower number of less than 25. In sum, global estimates on alcohol-attributable mortality and disease burden rely only on selected large disease categories. This eliminates most categories, which are 100 % alcohol-attributable by definition, except for alcohol use disorders and fetal alcohol syndrome (for some background on burden attached for alcohol use disorders: [39, 40]; for fetal alcohol syndrome [41]), as well as smaller partially attributable categories of disease or causes of death.

## Diseases and causes of death seen as causally impacted by alcohol

The number of alcohol-attributable disease categories in CRAs over the past decades has been increasing for three reasons. First, while the overall number is still small, more disease categories have been included into the global statistics (both as cause of death and as burden of disease in DALYs). To give two examples relevant for alcohol, pancreatitis and cardiac arrhythmias were added for the CRA of GBD study 2010 [17, 24]. Second, evidence on the causal impact of alcohol consumption became stronger and more convincing for certain disease categories, and third, better models for quantification of such causal impact were established.

### Alcohol-attributable cancers

For the second reason specified above, take alcohol-attributable cancers as an example [42]. While the first monograph from the meeting of the International Agency for Research on Cancer on alcohol use and cancer established sufficient evidence for a causal relationship between alcohol consumption and the cancer categories of nasopharyngeal cancer, esophageal cancer, laryngeal cancer, and liver cancer [43], the next meeting added female breast and colorectal cancers [44, 45]. The underlying evidence led to inclusion of breast cancer into the CRAs from the GDB study 2000 onwards, and for colorectal cancers from 2010 onwards. Currently other types of cancer are discussed as potentially alcohol-attributable, such as cancer categories of pancreas or stomach cancer, as heavy drinking has been consistently associated with increased risk for these categories [46, 47]; thus, more cancer categories will likely be added to future CRAs.

### Alcohol-attributable infectious diseases and causes of death

Of the alcohol-attributable disease categories, infectious diseases and causes of death constitute the most important overall change within the past two decades. Even though pneumonia and tuberculosis had been seen as impacted by heavy drinking as early as in the 18^th^ century [48], the causality had to be re-established using current criteria [49]. The first step was conducted in a consensus meeting in 2008, which established causality for tuberculosis and pneumonia [50]. This led to inclusion of these categories from 2010 onwards (underlying documentation: tuberculosis [51]; pneumonia [52]).

The open question was HIV/AIDS, where the meeting did not find enough evidence of causality despite consistent associations [53–55]. There was a clear causal association which could be quantified for one pathway: the impact of alcohol consumption on medication adherence, which had impact on mortality [56]. This association was implemented for the 2012 CRA, which was the basis of the WHO 2014 Global status report [9]. Moreover, in a number of recent systematic reviews based on experimental research, it could be established that alcohol has a causal impact on decisions to engage in unsafe sex [57, 58]. This allows for estimation of alcohol impacting the incidence of HIV as an additional component in future CRAs, and the responsible WHO technical advisory group has decided to include this component. This addition will markedly change the estimates of alcohol-attributable mortality and burden of disease in Sub-Saharan Africa [59].

### Alcohol use and mental disorders

It may be surprising that since 2010 no other mental disorders than alcohol use disorders have been included (i.e., alcohol dependence and the harmful use of alcohol according to ICD 10). Clearly most mental disorders have consistent associations with alcohol use, especially heavy drinking, and alcohol use disorders (e.g., [60–62]; we give only references for alcohol use disorders, as heavy drinking is very closely related to these disorders [63], and has even been suggested as a better definition for kind of disorders [64]). In addition to these associations, both DSM-5 and ICD 10 [65, 66] list alcohol-induced mental disorders, thus establishing causality. However, there is a problem in quantifying the causal impact. While alcohol consumption impacts mental disorders, there is also reverse causality, and we cannot exclude genetic vulnerability as a third variable impacting both alcohol consumption and mental disorders [16]. Thus, quantification of causality is difficult. It has been attempted in the GBD study 2000 for major depressive disorders [16], but later committees did not see this attempt as convincing enough. Maybe some of the newer research on alcohol and depression [67, 68] will allow for better modelling in future CRAs.

### Other alcohol-attributable disease and injury categories

Other categories of alcohol-attributable disease and mortality included in current CRAs are fetal alcohol syndrome (by definition), epilepsy [69], gastrointestinal disease (liver cirrhosis [70] and pancreatitis [37, 38]; the latter new for the CRA associated with GBD study 2010 and after), diabetes [71], cardiovascular disease [72] hypertensive disease [73], ischemic heart disease [74], stroke [75], and cardiac arrhythmias [76], the last one new for the CRAs 2010 and after, and almost all categories of injury [77, 78]. The last systematic overview on alcohol use and causal relations to different disease and cause of death categories can be found in [17].

The alcohol-attributable burden in all CRAs has almost entirely been estimated as the effect of drinking on the drinkers themselves. However, as with smoking, there is significant harm to others [79, 80] (for some first estimation within a CRA: [81]). In the CRAs thus far, only some of the effect of mothers’ drinking on newborns (in the last CRAs only fetal alcohol syndrome; before low birth weight – [82]) and unsystematically some of the effects on harm to others in traffic injuries have been captured.

In sum, with respect to changes of alcohol-attributable disease categories over time, there have been some categories added since 1990, mainly based on more disease categories available on a global basis. In addition, the evidence base has expanded in recent years to include and quantify the contribution of infectious disease categories as being alcohol-attributable. However, effects of alcohol consumption on others than the drinker have not been covered systematically.

## Relative risk estimates used to estimate attributable disease burden

Table 2 gives an overview on the relative risk estimates used for the WHO Global status report on alcohol and health [9] for all countries except for Russia and surrounding countries (for graphical displays of the dose-response relationships between average volume of consumption and outcomes see [83]; for the estimates used for Russia and surrounding countries see [84]).

**Table 2:** Categories of alcohol-attributable diseases and the sources used for determining risk relations from the WHO 2014 Global Status Report on Alcohol and Health [9]^a^

Condition	ICD 10 Code	Sources of risk relations (for calculating alcohol-attributable fractions)
Infectious and parasitic diseases		
Tuberculosis	A15-A19	[158]
Human immunodeficiency virus/ Acquired immune deficiency syndrome	B20-B24	[56] for estimate on the impact of alcohol on worsening the disease course via disrupting the medication schedule
Malignant neoplasms		
Mouth and oropharynx cancers	C00-C14	(based on relative risks from [87])
Esophageal cancer	C15	(based on relative risks from [87])
Liver cancer	C22	(based on relative risks from [87])
Laryngeal cancer	C32	(based on relative risks from [87])
Breast cancer	C50	(based on relative risks from [87])
Colon cancer	C18	(combined risk taken from [47])
Rectal cancer	C20	
Diabetes		
Diabetes mellitus	E10-E14	[71]
Neuro-psychiatric conditions		
Alcoholic psychoses (part of AUD)	F10.0, F10.3-F10.9	100 % alcohol attributable by definition
Alcohol abuse (part of AUD)	F10.1	
Alcohol dependence (part of AUD)	F10.2	
Accidental poisoning by and exposure to alcohol	X45	
Epilepsy	G40-G41	[69]
Cardiovascular disease		
Hypertensive disease	I10-I15	[73]
Ischemic heart disease	I20-I25	[89, 90, 159]
For any CRA after GBD 2013 see: [89]		
Cardiac arrhythmias	I47-I49	[76]
Ischemic stroke	I60-I62	[75, 89]
Hemorrhagic and other non-ischemic stroke	I63-I66	[75]
Digestive diseases		
Cirrhosis of the liver	K70, K74	[70]
Acute and chronic pancreatitis	K85, K86.1	[37]
Respiratory infections		
Lower respiratory infections	J10–J18, J20–J22	[52]
Conditions arising during the prenatal period		
Fetal alcohol syndrome	Q86.0	100 % alcohol attributable by definition
Unintentional injuries		
Motor vehicle accidents	^b^	[87]
Poisonings	X40-X49 except X45	[87]
Falls	W00-W19	[87]
Fires	X00-X09	[87]
Drowning	W65-W74	[87]
Other Unintentional injuries	^c^Rest of V-series and W20-W64, W 75-W99, X10-X39, X50-X59, Y40-Y86, Y88, Y89	[87]
Intentional injuries		[87]
Self-inflicted injuries	X60-X84, Y87.0	[87]
Homicide	X85-Y09, Y87.1	[87]

^a^Due to lack of data on very specific categories of death, diseases where alcohol is a necessary cause (other than Alcohol Use Disorders), such as alcohol poisonings, were modelled using RRs for the broader category

^b^V021–V029, V031–V039, V041–V049, V092, V093, V123–V129, V133–V139, V143–V149, V194–V196, V203–V209, V213–V219, V223–V229, V233–V239, V243–V249, V253–V259, V263–V269, V273– V279, V283–V289, V294–V299, V304–V309, V314–V319, V324–V329, V334–V339, V344–V349, V354–V359, V364–V369, V374–V379, V384–V389, V394–V399, V404–V409, V414–V419, V424–V429, V434–V439, V444–V449, V454–V459, V464– V469, V474–V479, V484–V489, V494–V499, V504–V509, V514–V519, V524–V529, V534–V539, V544–V549, V554–V559, V564–V569, V574–V579, V584–V589, V594–V599, V604–V609, V614–V619, V624–V629, V634–V639, V644–V649, V654– V659, V664–V669, V674–V679, V684–V689, V694–V699, V704–V709, V714–V719, V724–V729, V734–V739, V744–V749, V754–V759, V764–V769, V774–V779, V784–V789, V794–V799, V803–V805, V811, V821, V830–V833, V840–V843, V850– V853, V860–V863, V870–V878, V892

^c^Rest of V = V-series MINUS ^b^

While new meta-analyses on the risk relations between level of consumption and various disease/mortality outcomes appear regularly, this does not change the burden estimates dramatically. Take breast cancer as an example: there have been more than 100 single studies and 16 systematic reviews with meta-analyses over the past 20 years [85]. However, the main conclusions on relative risk did not change: there is a clear dose-response relationship with no protective effect for any level of drinking compared to lifetime abstainers [85]. Even drinking as low as one drink on average is associated with increased risk for breast cancer [85, 86]. The quantification for the different levels of alcohol-attributable risk for breast cancer had been quite similar over the years [85], as they had been for cancer in general (e.g., [87], which has been used to date for the CRAs, and [47]; which will be used in the future).

The field of cardiovascular outcomes has been less stable, in part, because two dimensions of alcohol consumption need to be taken into consideration, average volume and patterns of drinking, in particular heavy drinking occasions (in general: [88]; for cardiovascular in particular see [16, 89]), and because there are much fewer underlying studies (for some endpoints less than 10 studies; see the underlying studies on heavy drinking occasions and ischemic heart disease: [74, 90]), and because part of the effect is on acute drinking and part on chronic drinking with different methodologies which are hard to reconcile in meta-analytic approaches (acute drinking risks have been mainly measured via case-crossover studies such as [91]; chronic risks have been mainly measured in cohort studies such as [92]). Another problem is the fact that risk curves differ between fatal and non-fatal outcomes for many endpoints such as stroke [75] or ischemic heart disease [89]. Overall this makes the estimation of alcohol-attributable mortality and burden of disease challenging, and in almost each new CRA, a different approach has been used. As well, newest calculations in the WHO European Region suggest, that relatively small changes in exposure resulted in marked changes of cardiovascular mortality over the past 25 years [20].

Relative risks for other outcomes can be classified as in between cancer and cardiovascular disease in their complexity. For many outcomes, fatal and non-fatal risk relations differ. Thus, it has been found that for liver cirrhosis, the risk curves are steeper (more exponential) for mortality compared to non-fatal outcomes [70]. The explanation is simple: it takes quite a lot of alcohol consumption to cause liver cirrhosis often via different stages of liver disease [93], but once liver cirrhosis is established, no matter of what etiology, relatively small amounts of alcohol may be fatal [70, 94]. Heavy drinking occasions may play an additional role here, but we do not have enough data to quantify this relationship [95].

Similarly, for injury, acute alcohol use has been linked to more severe and in particular to fatal injuries [16, 96]. For CRA this means that different risk relations have to be used for mortality and for the non-fatal outcomes.

One of the main problems with the relative risks is that it is assumed, that they are biological constants and the same for all countries, and thus it does not matter, that the current estimates are derived from meta-analyses of select cohort studies from a few high income countries with a limited variation of drinking patterns (see above and [97], box on “Methodological issues relevant to studies of alcohol-related morbidity and mortality). Unfortunately, this assumption is not correct. It has been shown that the usual relative risks derived differ from the analogous risks found in Russia or surrounding countries [98, 99], and thus would lead to underestimates of alcohol-attributable burden [100]. In the most recent CRAs after 2010, this has been acknowledged and country specific relative risks have been used [84]. We suspect that different relative risks could be necessary for other countries with high *per capita* consumption per drinker (see [9], for a listing of countries), or for countries with irregular heavy-drinking patterns (e.g., countries with festive drinking such as Mexico; [101, 102]). Unfortunately, as of now, we have no evidence base to implement country- or region-specific relative risks for these patterns. Another problem is genetic predisposition, which sometimes interacts with alcohol consumption to produce different risks. As an example, the flushing gene [103], which is clearly associated with higher risks for alcohol-attributable cancer with an acetaldehyde pathway [104], clearly indicated different relative risk estimates for countries where this genetic constellation is prevalent (such as China, Japan and South Korea).

In sum, global estimates of risk relations tend to only minimally change for outcomes with many underlying studies such as cancer outcomes. This changes for cardiovascular outcomes, where more than one exposure dimension is relevant, and where there are few studies to quantify the risk relations. In future, more country- or region-specific relative risk estimates will be necessary to include genetic variability and more extreme drinking patterns, which are not measured in most cohort studies (see the box on “Methodological issues relevant to studies of alcohol-related morbidity and mortality” and [97] for further discussion of the limitations of cohort studies).

## Methodology used to derive attributable fractions

While the overall methodology for CRAs has been fairly stable (for a description see [105, 106]) based on original epidemiological concepts of the 1980s [107, 108], there are important shifts in details:

- The first CRAs until and including the 2010 study were based on discrete categories of exposure and associated relative risks, whereas the latter were based on a continuous distribution of both exposure and risk (for theoretical background: [109]; for a comparison of both methods in the same sample: [110])
- This implicated different ways to define exposure and to triangulate between national *per capita* consumption and surveys (see [16, 111] and [110, 112] for the categorical, and the continuous approach respectively). However, the differences between the categorical and the continuous approach for alcohol as a risk factor in CRAs are not that large [109].
- The biggest difference will come in via the triangulation of survey and *per capita* consumption. Overall *per capita* consumption is considered as most validly representing the overall consumption level in a population [113], especially if it is for countries with high proportion of recorded consumption ( [9] for the proportion of recorded to overall consumption). However, as *per capita* consumption is derived from administrative records [114], it allows no differentiation by sex and age. That is where surveys become indispensable. The problem is that general population surveys only cover part of the real consumption, and this proportion, usually labelled coverage rate [115, 116], is highly variable. The known variation of coverage rates is between 20 % and 90 % (for an example of low coverage: 27 % in Canada [117]; for high coverage see 87 % in Sweden [118] or close to 90 % in New Zealand [119]).
- So triangulation basically assumes the distribution by sex and age of the survey, and a consumption level of 80 % of the *per capita* consumption [110]. The reason for not assuming 100 % of *per capita* consumption which is sold is, that there is some spillage and other waste of alcohol, and that exposure is applied to risk relation estimates which may also underestimate consumption. However, the degree of underestimation is not clear, as there is no gold standard for such studies. There are some indications that cohort studies with their specific forms of assessment often in a medical environment tend to show less underestimation of true consumption [120–122]. Moreover, lack of coverage does not only reflect individuals underestimating or misrepresenting their consumption. It also reflects the sample frame of the survey, which usually excludes high drinking populations such as military or institutionalized people [123]. As a result it is hard to calibrate a certain proportion of *per capita* consumption, but the WHO technical advisory committee after reviewing all the underlying evidence decided for 80 %. The impact of different triangulations can be considerable [117, 124], so it is good to be conservative [125].

## Implications for research

In the above, we described the methodology to conduct a CRA for alcohol consumption. In this methodology, instantaneous effects are assumed: i.e., exposure to alcohol consumption in a certain year is assumed to result in changes in mortality for this year. This clearly is a simplification, as there is usually a lag time between alcohol consumption and disease outcomes [126]. Moreover, individual consumption may vary, and many risk relations assume more or less a constant consumption over time [127]. Future CRAs should address this problem and take into consideration the lag time between consumption and outcomes (e.g., using methodology such as [128, 129]). However, such a step would need a reconfiguration of the conceptual model for all CRAs for all risk factors, as one of the main objectives of any CRA is to be comparative between risk factors and time.

In addition we expect the following methodological changes for future CRAs for alcohol:

- With respect to modelling exposure, methods to triangulate irregular heavy drinking occasions with *per capita* consumption are needed. As indicated above, currently the only triangulation is between average level of alcohol consumption and *per capita* consumption [110, 112]. For irregular heavy drinking occasions [130, 131], we accept self-reports from surveys as true, even if we know, that they usually underestimate true frequency and number of drinks per occasion. In the future, we need to develop ways to triangulate self-report and objective measurement for irregular heavy drinking occasions.
- We have already mentioned the rather strong assumption, that risk relations taken from the most comprehensive meta-analysis are seen as a global constant with exception of Russia and surrounding countries (where the risk relations are based on [98, 99]; see also [84]). Given the genetic and environmental differences, we would expect some differences in risk relations between alcohol consumption and disease/mortality outcomes in different regions (e.g. based on the interaction between genetics and alcohol consumption in causing cancer; see [104, 132]). Future CRAs for alcohol consumption will have to more and more regionalize risk relations, not only based on genetic predisposition, but also based on socially determined risks such as the risks for injury outcomes [133, 134].
- Finally, we expect that future CRAs will have explicit separation on harm to drinkers and harm to others. The conceptual framework is there [135], the underlying data for major categories such as traffic injury [136] or for fetal alcohol syndrome/fetal alcohol spectrum disorders is there (the latter estimated via drinking in pregnant women; [137, 138]) and there are major efforts to improve the methodology for quantification (e.g., in the Netherlands based on the *per se* law on substance use and violence – [139]; in Germany within a comprehensive effort to estimate alcohol-attributable harm to others for the Ministry – personal communication of Prof. L. Kraus).

## Implications for alcohol policy

As indicated above, all CRAs resulted in marked burden of disease caused by alcohol consumption. Two dimensions were identified as important to cause harm: overall level of consumption and patterns of drinking [88]. Policies need to address both dimensions.

There are effective and cost-effective policies to lower overall level of consumption in societies [140, 141], such as the so-called ‘best buys’, i.e., increase of taxation leading to increases in price of alcoholic beverages, decrease in availability, and ban of marketing and advertisement [142, 143]. However, these policies do not to seem to be too popular with governments, and in fact alcohol has become more available and affordable in most parts of the world over the past decades (e.g., in the European Union, see [144]). Other potential ways to decrease overall level of alcohol consumption would be a decrease in alcoholic strength, which is technically possible for all beverages, and which could be achieved via government regulation, taxation or industry initiatives [145]. It should be noted however, that a reduction in overall level of alcohol consumption does not necessarily mean a reduction in total alcohol-attributable mortality or burden of disease, as monitoring of the last 25 years for the WHO European Region has shown [20]. In addition, it has to be assured, that the heaviest drinkers do not increase their drinking (e.g., via treatment [146]), and that patterns of drinking do not get worse (see also [20]).

Regarding patterns of drinking, there are other promising policies such as minimum pricing [147, 148], and specific policies to decrease heavy drinking occasions in certain situation, such as in participation in traffic [136] or while operating machinery at the workplace [149]. Obviously, harm would be minimized, if in such situations abstinence was the norm.

Finally, the composition of alcohol-attributable burden of disease and mortality will have different implications for policy [150]. A high proportion of traffic injury could be reduced with specific measures for drink driving such as introduction and enforcement of a *per se* law regarding blood alcohol concentration, or reduction of the blood alcohol concentration threshold in existing laws [140, 151]. On the other hand, high alcohol-attributable intentional injury will ask for specific measures such as measures against binge drinking or *per se* laws on criminal prosecution [150]. To give one final example concerning chronic disease: high levels of alcohol-attributable liver disease mortality point to high overall level of consumption [152, 153], or to relatively high level of consumption combined with other etiological factors for liver disease such as HIV (as even comparatively small levels of alcohol consumption may cause liver mortality in people with liver cirrhosis no matter which etiology – see above for further detail and [20], for examples). Reductions of overall alcohol consumption, no matter how achieved, will lead to reductions in alcohol-attributable liver mortality [20].

## Conclusions

The CRA methodology has been evolving and for comparisons over time it is necessary to use the latest methodology and calculate backwards using the same methodology. If this principle is used, then CRAs can potentially inform the health policy process and yield important information for decision makers. Obviously, interventions will depend not only on the size and shape of the burden, but also on how much of the alcohol-attributable burden is avoidable [154], and on aspects on feasibility, costs and cost-effectiveness of interventions [14, 15]. For alcohol consumption, in principle all of the burden is avoidable, but any intervention will have to take into consideration the role alcohol has been playing in our society for thousands of years [13]. However, despite these general limitations, information about attributable burden will also be one major building block towards better policies [19, 150].

## Abbreviations

AIDS: Acquired Immune Deficiency Syndrome
AUD: Alcohol Use Disorder
CRA: Comparative Risk Assessment
DALYs: Disability Adjusted Life Years
DSM: Diagnostic and Statistical Manual of Mental Disorders
GBD: Global Burden of Disease
HIV: Human Immunodeficiency Virus
ICD: International Classification of Disease
IHME: Institute for Health Metrics and Evaluation
WHO: World Health Organization
